# Supplementary material for: Bicontinuous RuO2 nanoreactors for acidic water oxidation
Source: Nat Commun. 2024 May 9;15:3928. doi: 10.1038/s41467-024-48372-4 (PMC11082236; doi:10.1038/s41467-024-48372-4)
Supplement: Supplementary file 1 — Supplementary Information [file 41467_2024_48372_MOESM1_ESM.pdf]

Supplementary Information for

Bicontinuous RuO_2 nanoreactors for acidic water oxidation

Ding Chen[1], Ruohan Yu[1, 2], Kesong Yu[1], Ruihu Lu[1], Hongyu Zhao[1], Jixiang Jiao[1], Youtao Yao[1], Jiawei Zhu[1], Jinsong Wu[1, 3], Shichun Mu[*] [1]

[1]*State Key Laboratory of Advanced Technology for Materials Synthesis and Processing, Wuhan University of Technology, Wuhan 430070, China.*

[2]*The Sanya Science and Education Innovation Park of Wuhan University of Technology, Sanya 572000, China.*

[3]*NRC (Nanostructure Research Centre), Wuhan University of Technology, Wuhan 430070, China.*

* *Corresponding author E-mail: msc@whut.edu.cn*

Content

Figure S1. The phase diagram of the two-salt system (KCl + LiCl), which coming from the http://www.crct.polymtl.ca/FACT/documentation/ (FTsalt → KCl-LiCl). 5

Figure S2. The digital photography of as-prepared MD-RuO_2-BN. 6

Figure S3. HAADF-STEM images (a, b) and the corresponding BF images (c, d) of MD-RuO_2-BN. 7

Figure S4. Gaussian fitted size distribution of RuO_2 nanoparticles. 8

Figure S5. (a) HAADF-STEM image, (b) BF-STEM image, and the corresponding elemental distribution of (c) Ru and (d) O for MD-RuO_2-BN. 9

Figure S6. Crystal structure of RuO_2 (x, y, z=1×1×1) from different perspectives. 10

Figure S7. Representative high-resolution STEM image of MD-RuO_2-BN. 11

Figure S8. STEM images with progressively higher resolution of commercial RuO_2. The scale bars are (a) 200 nm, (b) 100 nm, (c) 20 nm, and (d) 5 nm, respectively. 12

Figure S9. The reconstructed MD-RuO_2 nanoparticles viewed from different perspectives. 13

Figure S10. The extracted cubic sub volume viewed from different perspectives. 14

Figure S11. The front and top view of sub volume in figure 2f. 15

Figure S12. (a-c) The volumes extracted from high contrast segmentation. (d-h) The corresponding sectional view of different positions inside. 16

Figure S13. (a-c) The volumes extracted from low contrast segmentation. (d-h) The corresponding sectional view of different positions inside. 17

Figure S14. XRD pattern of K-RuO_2. K-RuO_2 is the products obtained from a blank experiment using KCl instead of KCl-LiCl eutectic system as a control under the same conditions. 18

Figure S15. (a-c) STEM images of K-RuO_2. (d) Corresponding high-resolution atomic image from the area indicated by the yellow box in figure (c). (e-h) STEM mapping and the corresponding elemental distribution of K-RuO_2. 19

Figure S16. STEM images of (a) MD-RuO_2-BN, (b) K-RuO_2, and (c) C-RuO_2. 20

Figure S17. 70 STEM-HAADF images of K-RuO_2 for tomography reconstruction. 21

Figure S18. The resulting tomography reconstructed structural unit of K-RuO_2, which was obtained from 70 STEM-HAADF images were collected by a 1-2° interval. 22

Figure S19. (a-c) Corresponding front, top and right view of reconstructed K-RuO_2. (d) Extracted cubic sub volume from the labeled red dash line area in figure (a-c). (e) The right view of sub volume. (f) Representative ortho slices marked by black dash line in figure (e). (g) Volumes from segmentation by contrast corresponding to RuO_2 (blue) and void (black), respectively. 23

Figure S20. Schematic illustration of the synthesis of the RuO_2 bicontinuous nanoreactors. . 24

Figure S21. Representative atomic STEM image of MD-RuO_2-BN, where the positions marked by yellow circles indicate the Ru vacancies present in the catalyst. 25

Figure S22. Representative atomic HAADF-STEM (a, b) and corresponding BF-STEM (c, d) images of MD-RuO_2-BN. 26

Figure S23. Ru K-edge EXAFS (points) and fit (line) for Ru powder (a), C-RuO_2 (c), and MD-RuO_2-BN (e), shown in k^2 weighted *R*-space. Ru K-edge EXAFS (points) and fit (line) for Ru powder (b), C-RuO_2 (d), and MD-RuO_2-BN (f), shown in k^2 weighted *k*-space. 27

Figure S24. (a-d) Photographs of pH meter for measuring 0.5 M H_2SO_4. (e) Corresponding column chart. 28

Figure S25. Cyclic voltammograms of (a) MD-RuO_2-BN, (c) C-RuO_2 and (e) K-RuO_2 in the region of (0.70) - (0.80) V versus SCE at different scan rates. Corresponding linear relationships between capacitive current and scan rate of (b) MD-RuO_2-BN, (d) C-RuO_2 and (f) K-RuO_2. 29

Figure S26. The surface energy of RuO_2, RuO_2-V_O, RuO_2-V_{Ru}, RuO_2-T and RuO_2-T-$V_{Ru,\ O}$. 30

Figure S27. The contact angles of MD-RuO_2-BN (45°) and C-RuO_2 (60°). The reduced contact angle here indicating the generation of various defects increase the surface energy of MD-RuO_2-BN and determine the strong hydrophilicity. 31

Figure S28. The accelerated degradation measurements of MD-RuO_2-BN for OER. 32

Figure S29. (a) XRD pattern and (b) XPS survey of MD-RuO_2-BN before and after OER electrolysis in 0.5 M H_2SO_4. 33

Figure S30. STEM images of MD-RuO_2-BN after OER electrolysis in 0.5 M H_2SO_4. (a) STEM-HAADF image. (b) Corresponding STEM-BF image. (c-d) STEM images and corresponding EDX elemental maps for Ru (e) and O (f). 34

Figure S31. The experimental set-up of ICP-OES. Photographs of (a) the conventional three-electrode system, (b) the electrochemical testing system, (c) the ICP-OES (700 Series, Agilent Technologies). (d) Photographs of electrolytes collected by step-by-step (1-1~1-7 for MD-RuO_2-BN; 2-1~2-7 for K-RuO_2), the time interval was 1 day (24 h), a total of 7 days. 35

Figure S32. The obtained standardization curve of Ru standard solution. For ICP-OES studies, we first taken 0, 0.25, 0.5, 1.0, 2.5 mL of 100 mg/L Ru standard solution, respectively, into a 50 mL volumetric bottle, then add 5% dilute nitric acid to steady volume to 50 mL and shake well to obtain above standardization curve 36

Figure S33. MD-RuO_2-BN || Pt/C and C-RuO_2 || Pt/C toward overall water splitting. Inset: the device of water splitting. 37

Figure S34. (a) Scheme of the two-electrode cell. (b) Gas collection device of water splitting. Photographs of oxygen (c) and hydrogen (d) collected at different times. 38

Figure S35. Amount of H_2 and O_2 in water electrolysis as a function of time....................... 39

Figure S36. (a) Photographs of the PEMWE testing system, including electrochemical workstation, electrolyzer, thermocouple, peristaltic pump and water tank. (b, c) Enlarged close-up of core component electrolyzer from different perspectives..40

Figure S37. (a) Photograph of pressure-sensitive paper. (b) Surface stress distribution nephogram of PTL and pressure-sensitive paper. 41

Figure S38. (a) Cross-sectional and (b) planar SEM images of MD-RuO_2-BN coated membrane. 42

Figure S39. Photos of the electrodes before (a) and after (b) PEMWE testing. 43

Figure S40. (a) Cross-sectional and (b) planar SEM images of MD-RuO_2-BN coated membrane after PEMWE testing. 44

Figure S41. Demonstrate the use of hydrogen produced from PEMWE to drive hydrogen fuel cell...45

Figure S42. Top views of the *OOH, *OH, *O adsorption configuration on routine Ru sites for RuO_2 (a), RuO_2-V_O (b), RuO_2-V_{Ru} (c), RuO_2-T (d), and RuO_2-T-$V_{Ru, O}$ (e). The white, red, and indigo balls represent the H, O, and Ru atoms, respectively. The black circles and yellow highlights denote the active sites and vacancy, respectively. 46

Figure S43. The free energy of OER critical intermediates including OH*, O*, OOH* on routine Ru site for RuO_2, RuO_2-V_O, RuO_2-V_{Ru}, RuO_2-T, and RuO_2-T-$V_{Ru, O}$.........................47

Figure S44. Top views of the *OOH, *OH, *O adsorption configuration on TB-Ru sites for RuO_2-T-V_O (a), RuO_2-T-V_{Ru} (b), and RuO_2-T-$V_{Ru, O}$ (c). The white, red, and indigo balls represent the H, O, and Ru atoms, respectively. The black circles and yellow highlights denote the active sites and vacancy, respectively..48

Figure S45. Photographs of experimental set-up of in-situ Raman (Horiba LabRAM HR Evolution)...49

Table S1. Comparison of OER performance of MD-RuO_2-BN with recently reported Ru-based electrocatalysts at 10 mA cm^{-2} in acidic media..50

Table S2. ICP investigation at regular intervals (24 h) during OER processes catalyzed by MD-RuO_2-BN and K-RuO_2, respectively. 51

References: 52

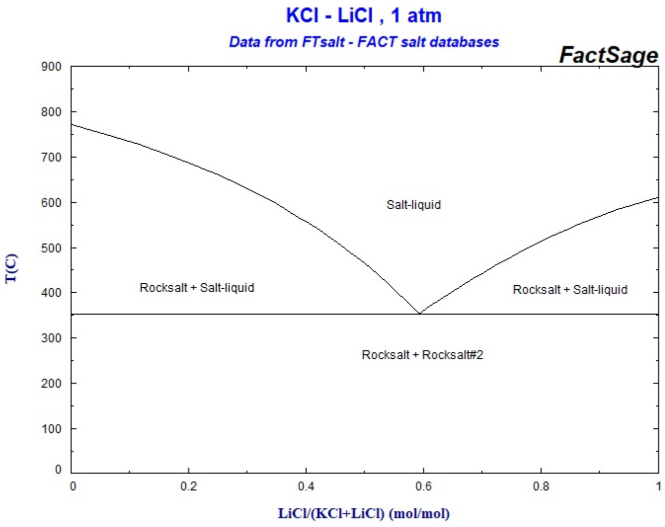

Figure S1. The phase diagram of the two-salt system (KCl + LiCl), which coming from the http://www.crct.polymtl.ca/FACT/documentation/ (FTsalt → KCl-LiCl).

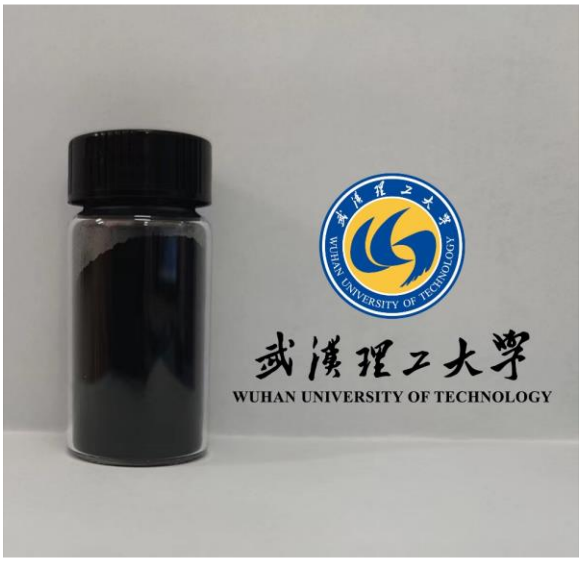

Figure S2. The digital photography of as-prepared MD-RuO_2-BN.

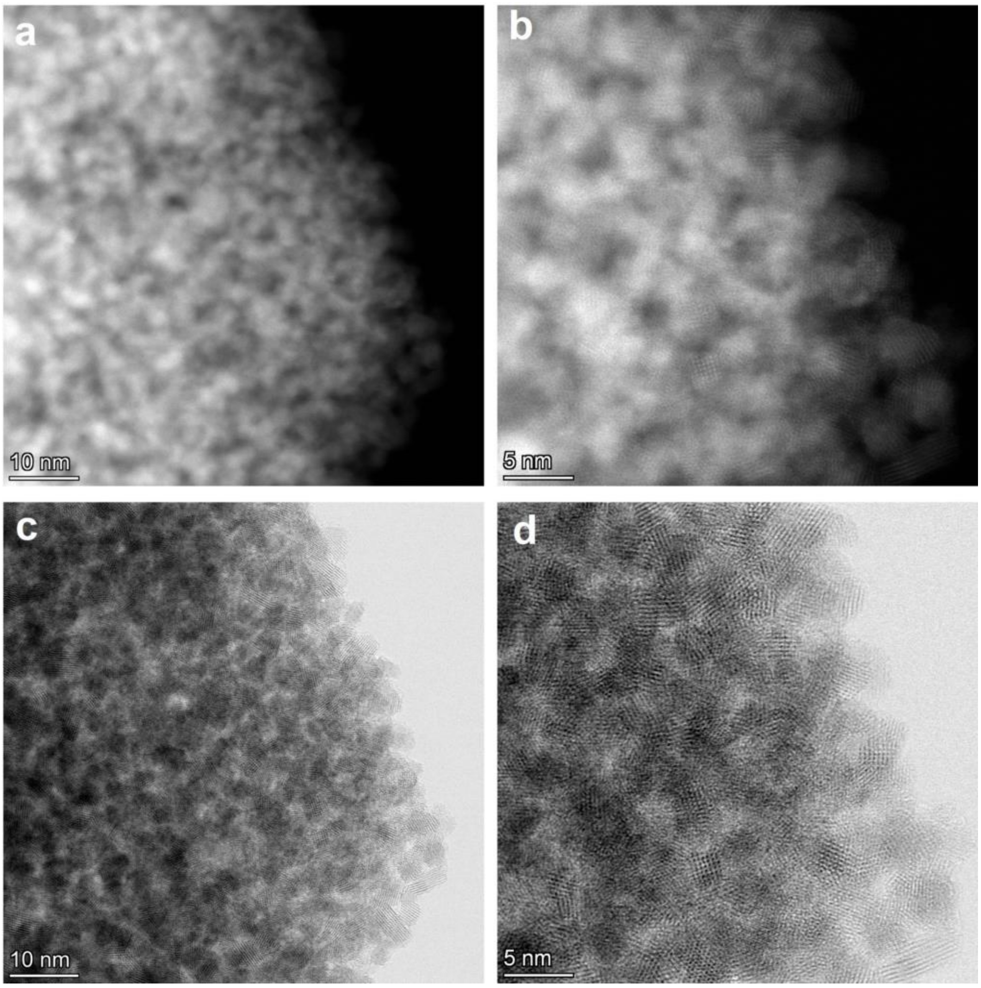

Figure S3. HAADF-STEM images (a, b) and the corresponding BF images (c, d) of MD-RuO_2-BN.

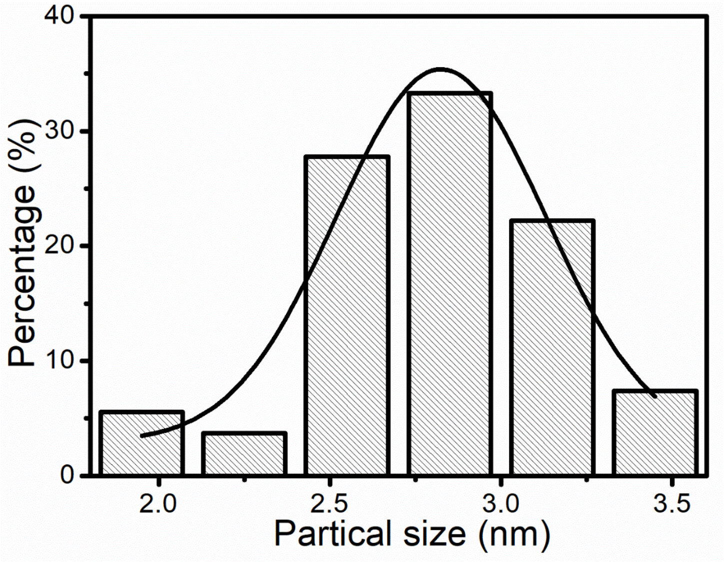

Figure S4. Gaussian fitted size distribution of RuO_2 nanoparticles.

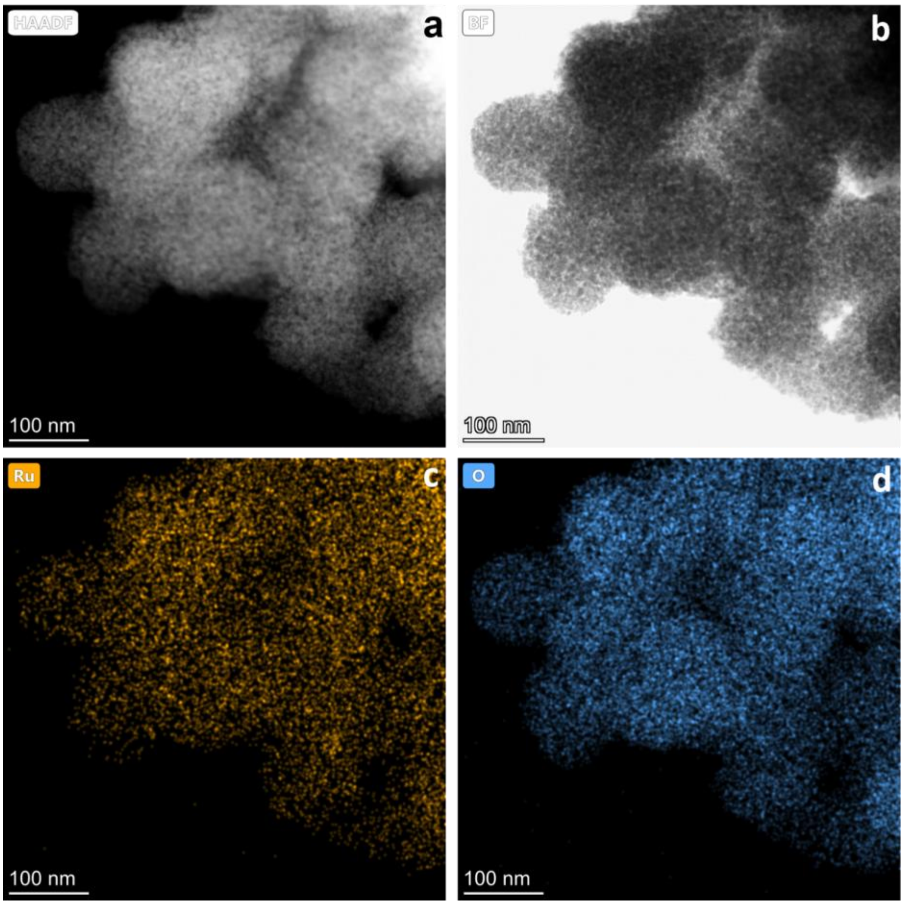

Figure S5. (a) HAADF-STEM image, (b) BF-STEM image, and the corresponding elemental distribution of (c) Ru and (d) O for MD-RuO_2-BN.

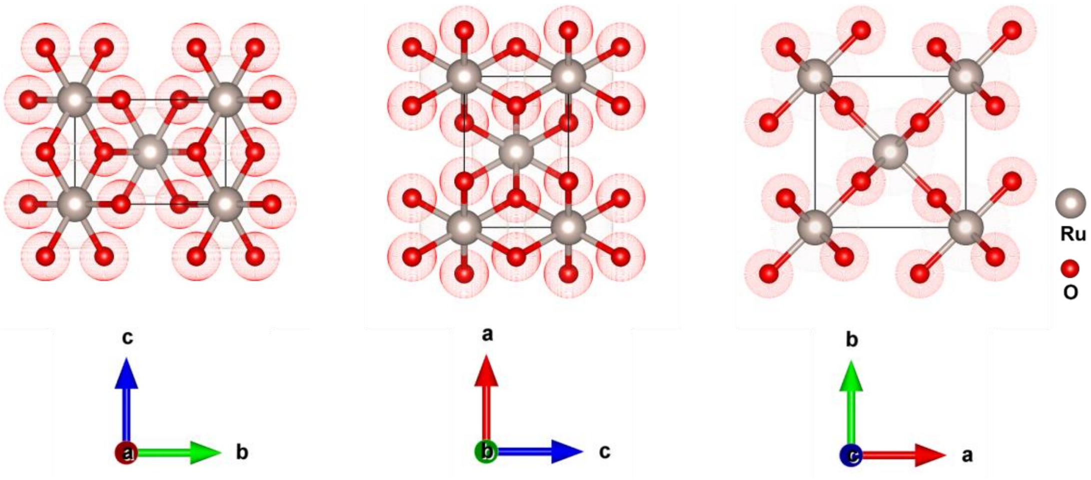

Figure S6. Crystal structure of RuO_2 (x, y, z=1×1×1) from different perspectives.

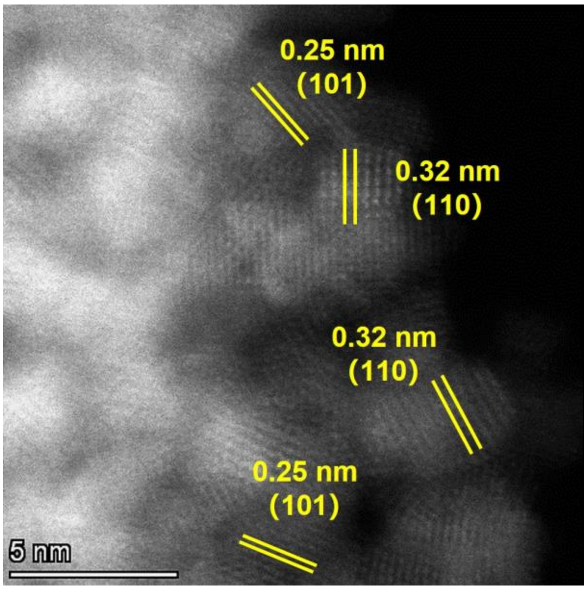

Figure S7. Representative high-resolution STEM image of MD-RuO_2-BN.

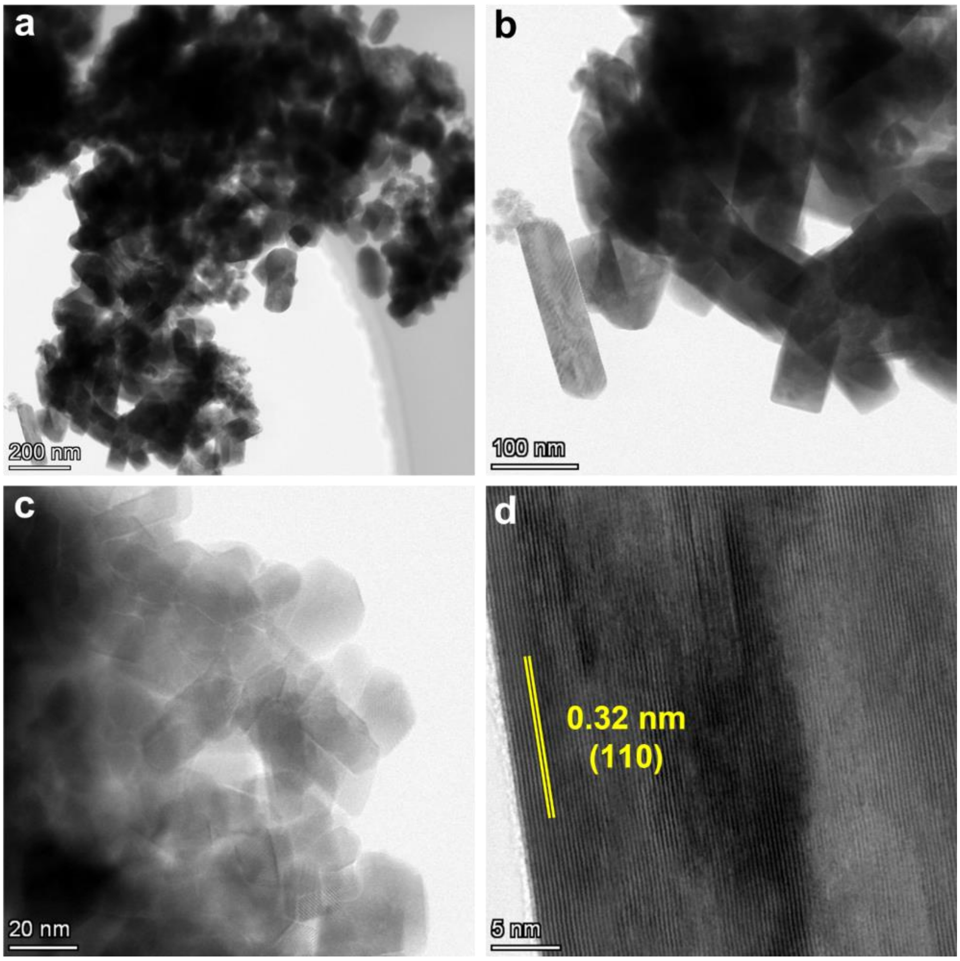

Figure S8. STEM images with progressively higher resolution of commercial RuO_2. The scale bars are (a) 200 nm, (b) 100 nm, (c) 20 nm, and (d) 5 nm, respectively.

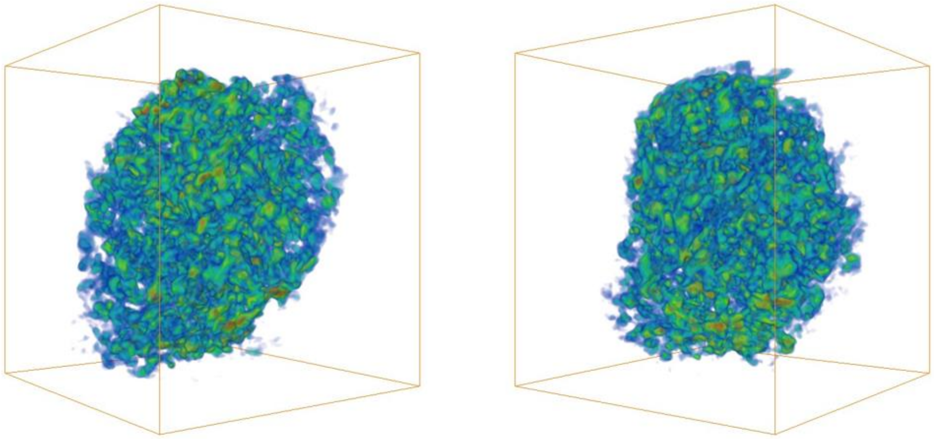

Figure S9. The reconstructed MD-RuO_2 nanoparticles viewed from different perspectives.

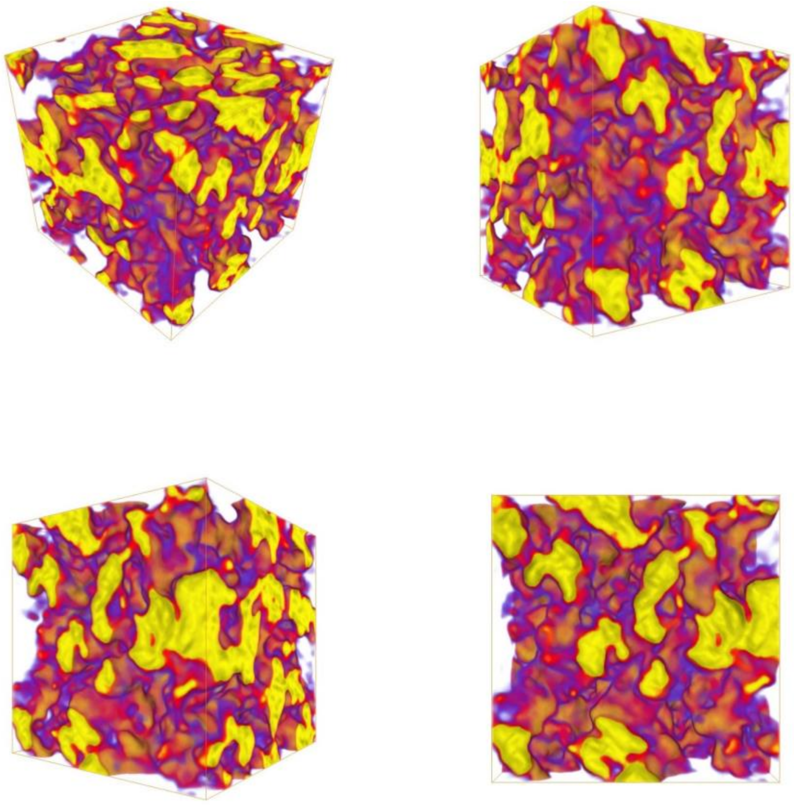

Figure S10. The extracted cubic sub volume viewed from different perspectives.

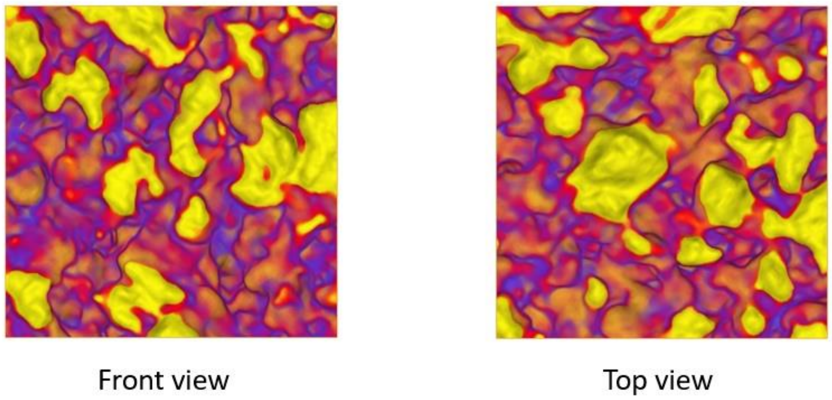

Figure S11. The front and top view of sub volume in figure 2f.

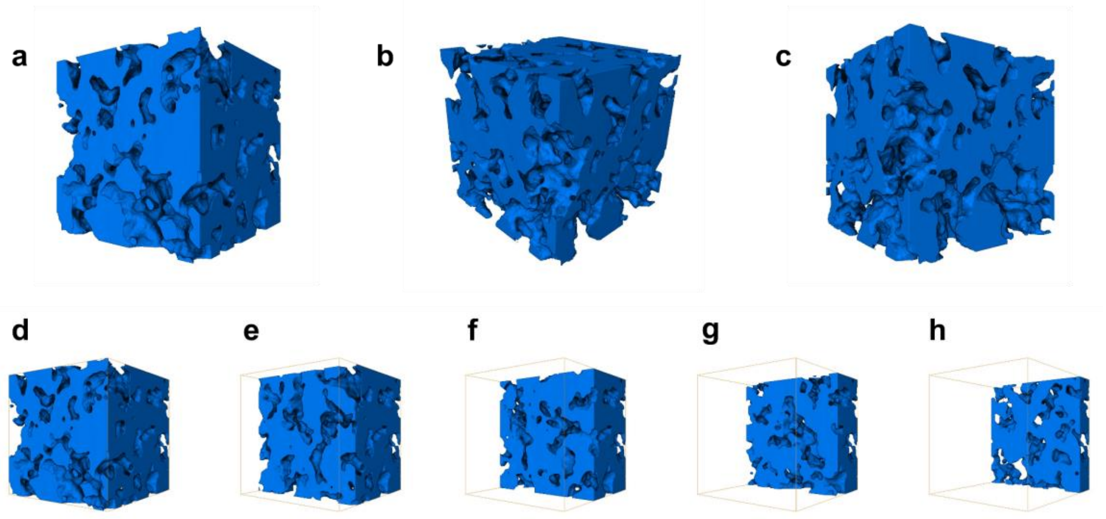

Figure S12. (a-c) The volumes extracted from high contrast segmentation. (d-h) The corresponding sectional view of different positions inside.

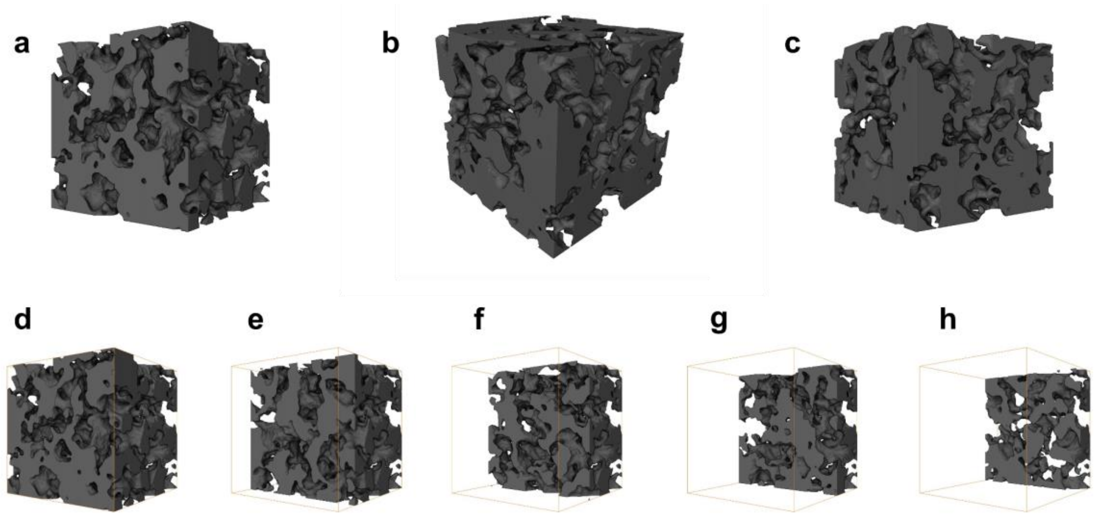

Figure S13. (a-c) The volumes extracted from low contrast segmentation. (d-h) The corresponding sectional view of different positions inside.

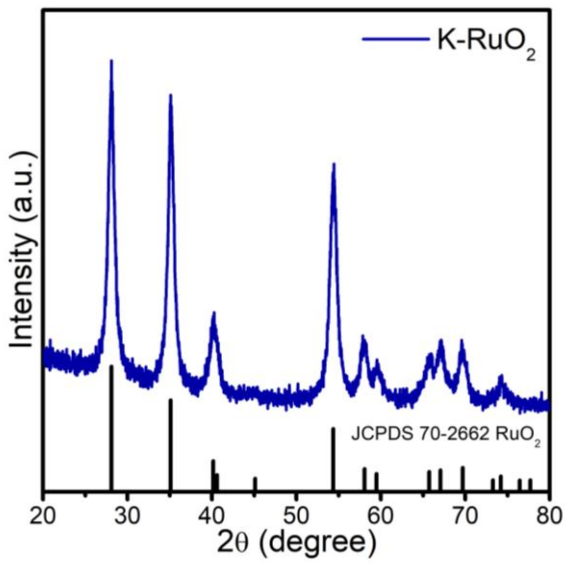

Figure S14. XRD pattern of K-RuO_2. K-RuO_2 is the products obtained from a blank experiment using KCl instead of KCl-LiCl eutectic system as a control under the same conditions.

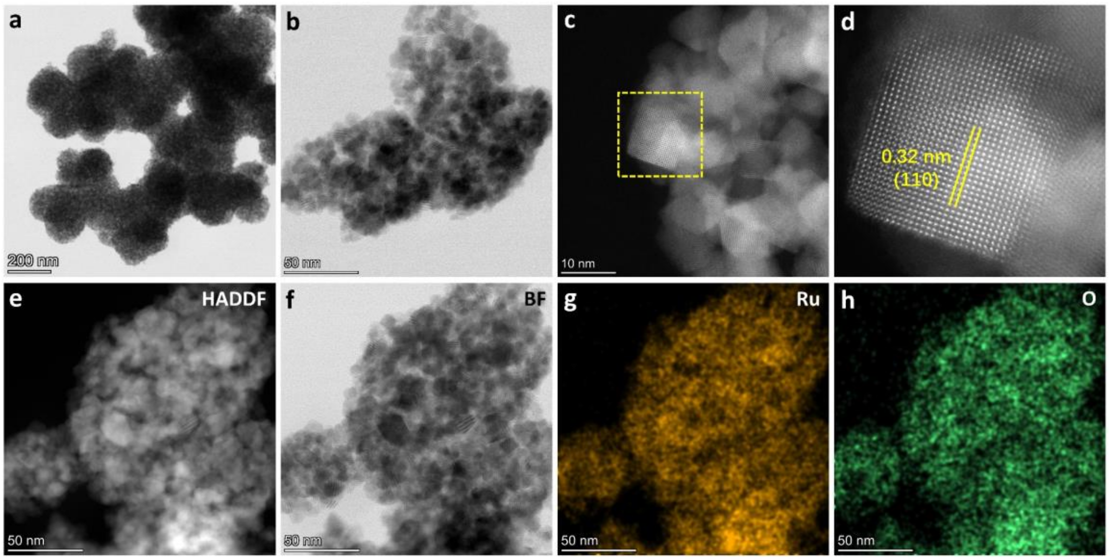

Figure S15. (a-c) STEM images of K-RuO_2. (d) Corresponding high-resolution atomic image from the area indicated by the yellow box in figure (c). (e-h) STEM mapping and the corresponding elemental distribution of K-RuO_2.

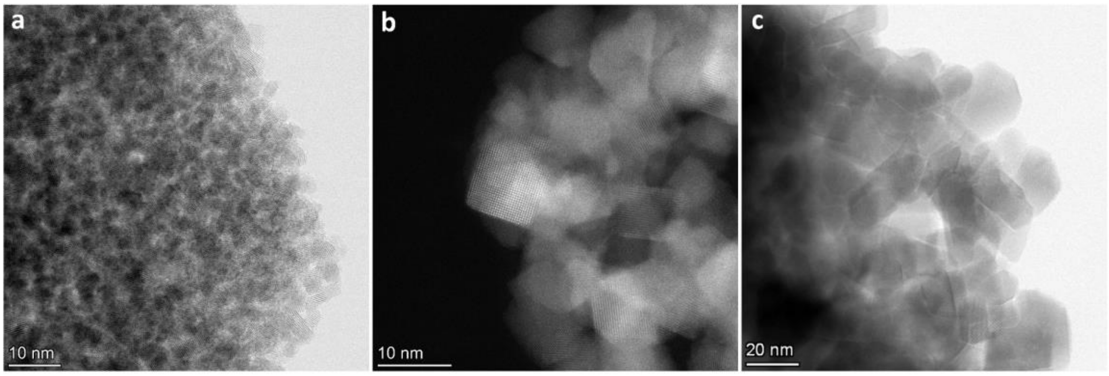

Figure S16. STEM images of (a) MD-RuO_2-BN, (b) K-RuO_2, and (c) C-RuO_2.

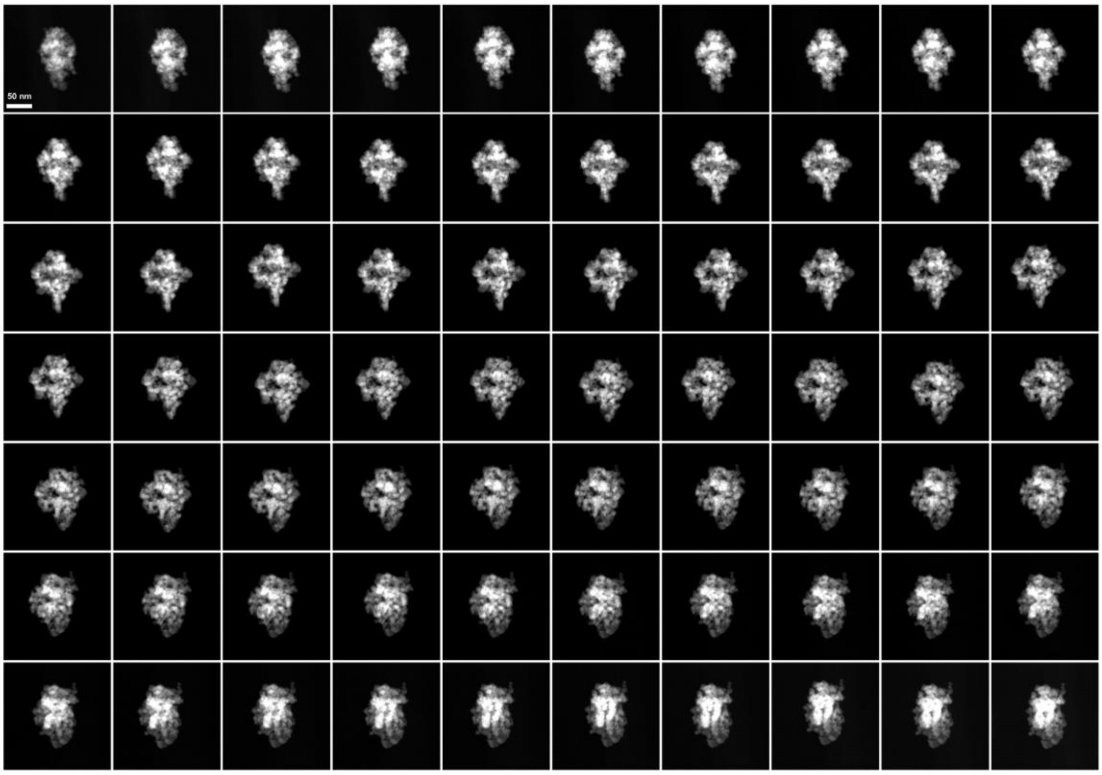

Figure S17. 70 STEM-HAADF images of K-RuO_2 for tomography reconstruction.

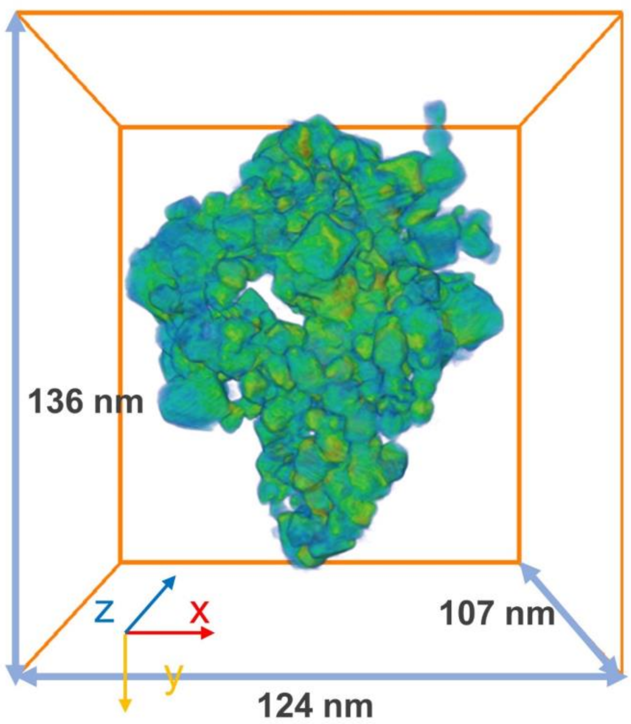

Figure S18. The resulting tomography reconstructed structural unit of K-RuO_2, which was obtained from 70 STEM-HAADF images were collected by a 1-2° interval.

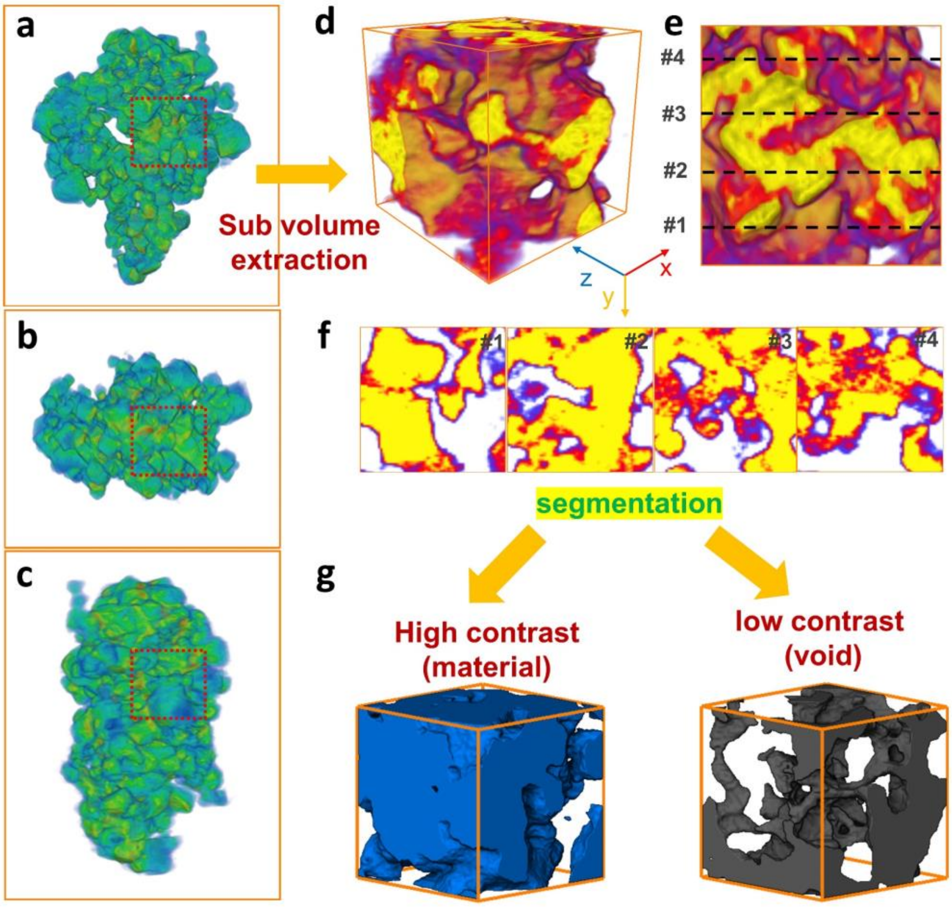

Figure S19. (a-c) Corresponding front, top and right view of reconstructed K-RuO_2. (d) Extracted cubic sub volume from the labeled red dash line area in figure (a-c). (e) The right view of sub volume. (f) Representative ortho slices marked by black dash line in figure (e). (g) Volumes from segmentation by contrast corresponding to RuO_2 (blue) and void (black), respectively.

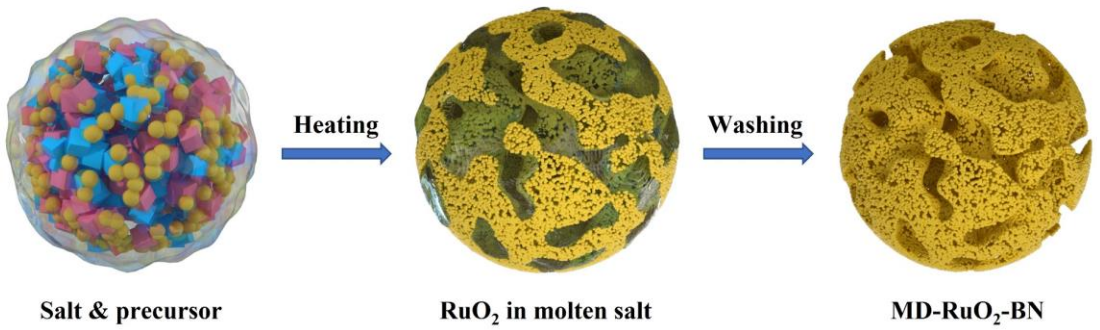

Figure S20. Schematic illustration of the synthesis of the RuO_2 bicontinuous nanoreactors.

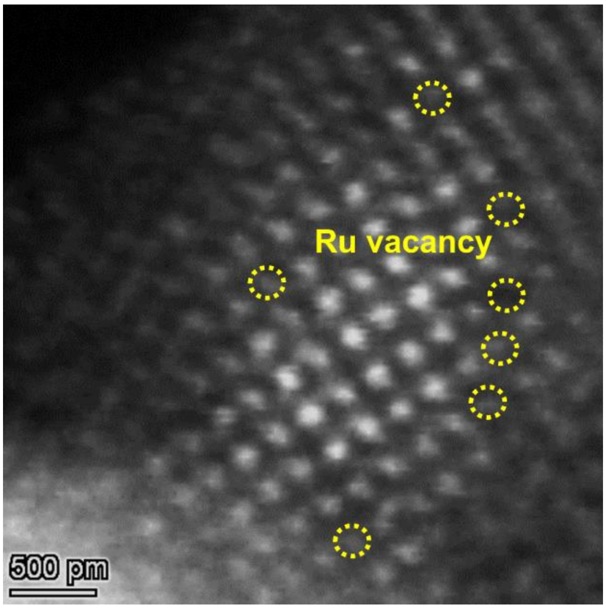

Figure S21. Representative atomic STEM image of MD-RuO_2-BN, where the positions marked by yellow circles indicate the Ru vacancies present in the catalyst.

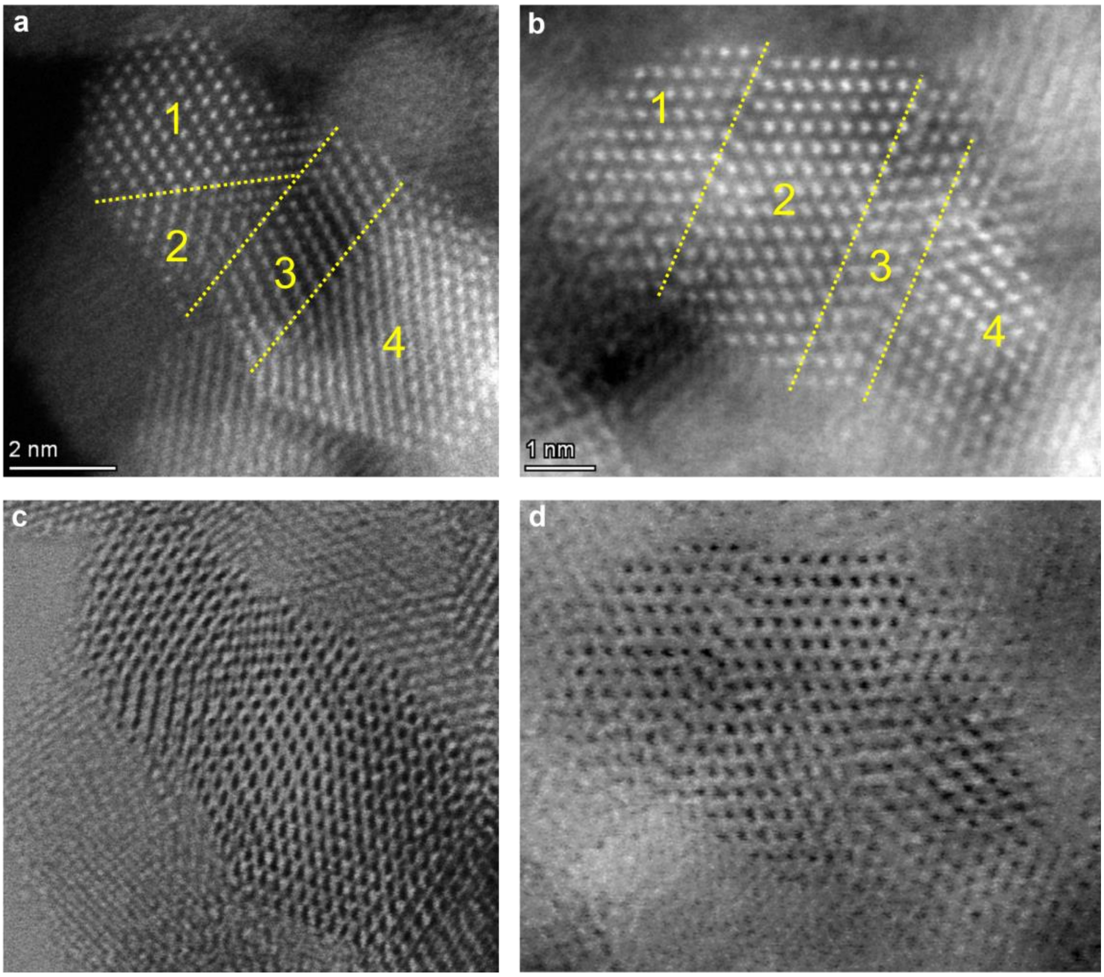

Figure S22. Representative atomic HAADF-STEM (a, b) and corresponding BF-STEM (c, d) images of MD-RuO_2-BN.

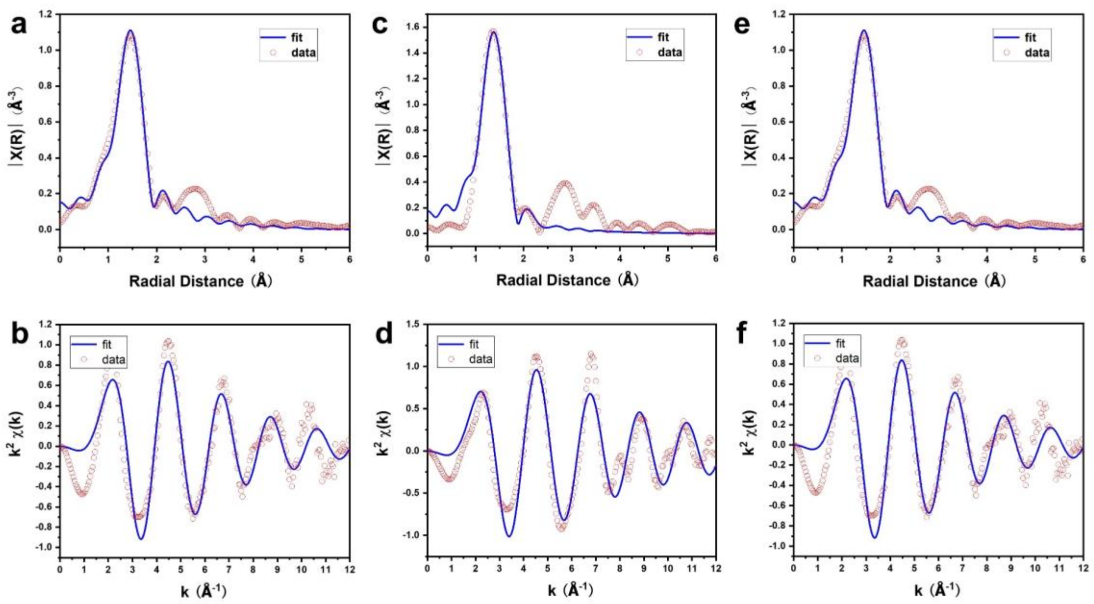

Figure S23. Ru K-edge EXAFS (points) and fit (line) for Ru powder (a), C-RuO_2 (c), and MD-RuO_2-BN (e), shown in k^2 weighted *R*-space. Ru K-edge EXAFS (points) and fit (line) for Ru powder (b), C-RuO_2 (d), and MD-RuO_2-BN (f), shown in k^2 weighted *k*-space.

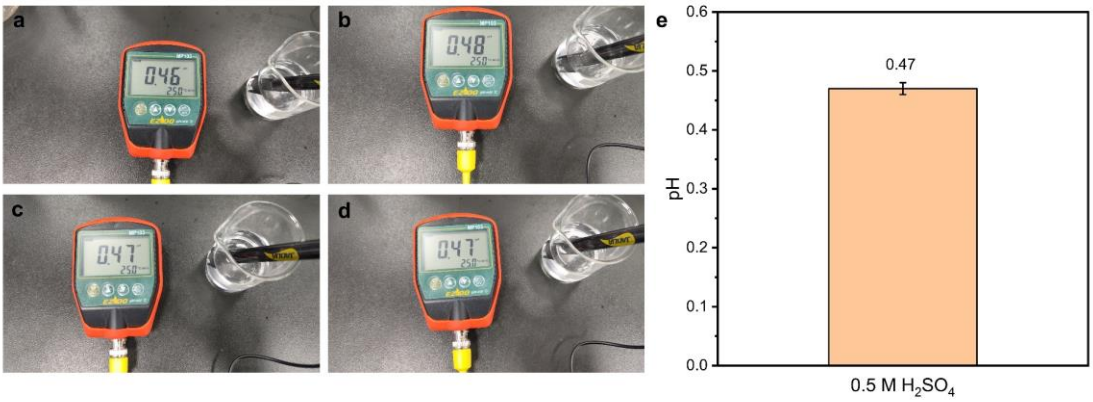

Figure S24. (a-d) Photographs of pH meter for measuring 0.5 M H_2SO_4. (e) Corresponding column chart.

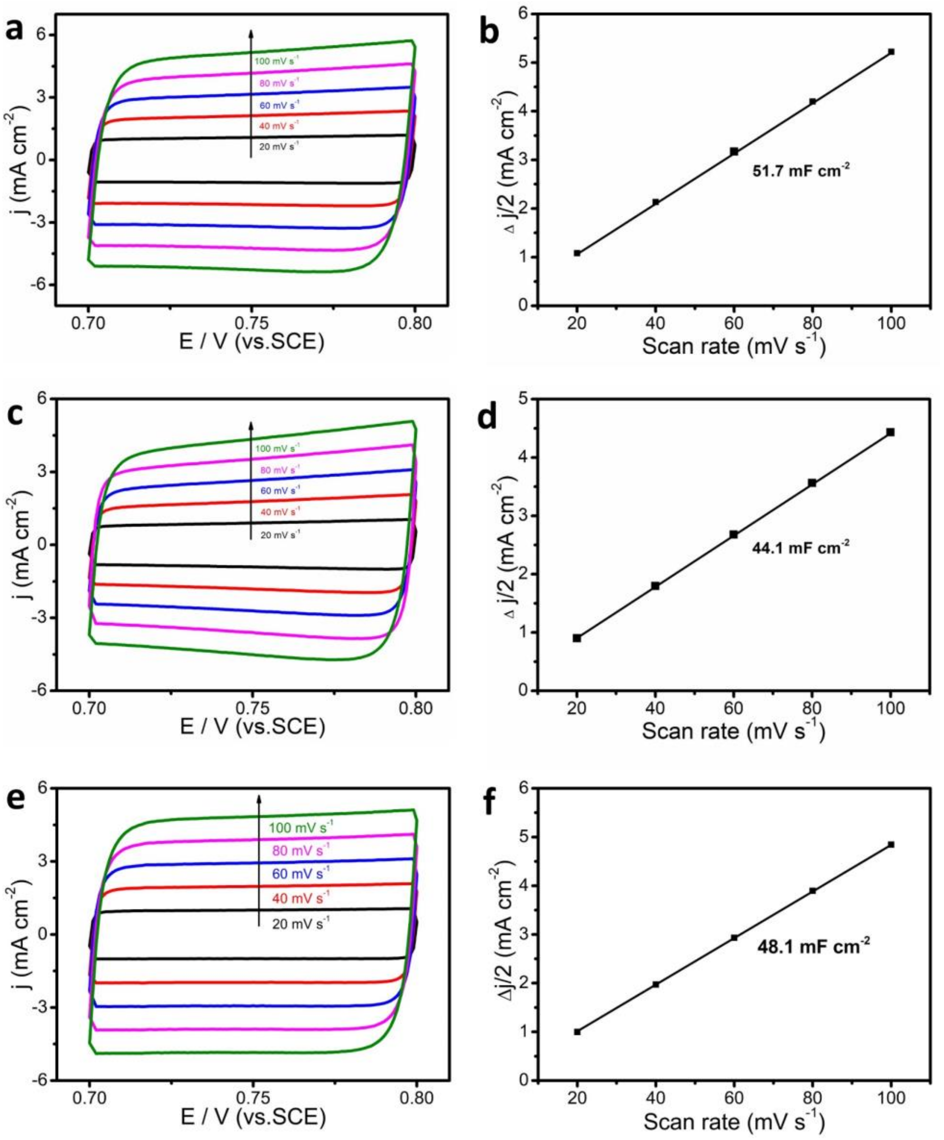

Figure S25. Cyclic voltammograms of (a) MD-RuO_2-BN, (c) C-RuO_2 and (e) K-RuO_2 in the region of (0.70) - (0.80) V versus SCE at different scan rates. Corresponding linear relationships between capacitive current and scan rate of (b) MD-RuO_2-BN, (d) C-RuO_2 and (f) K-RuO_2.

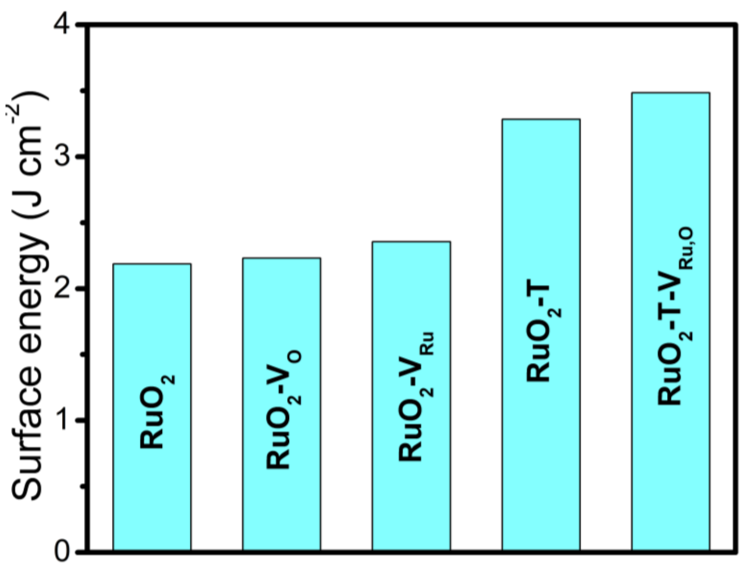

Figure S26. The surface energy of RuO_2, RuO_2-V_O, RuO_2-V_{Ru}, RuO_2-T and RuO_2-T-$V_{Ru, O}$.

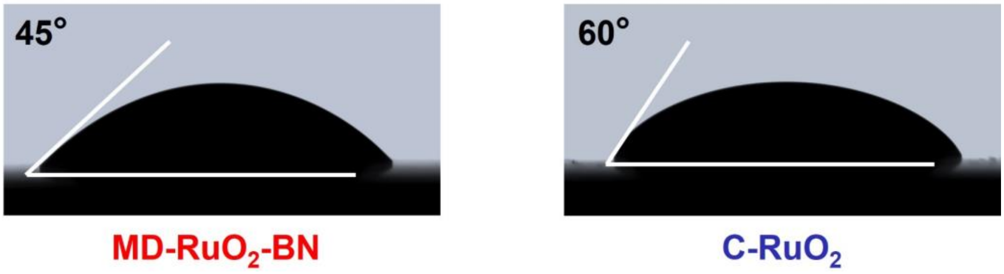

Figure S27. The contact angles of MD-RuO_2-BN (45°) and C-RuO_2 (60°). The reduced contact angle here indicating the generation of various defects increase the surface energy of MD-RuO_2-BN and determine the strong hydrophilicity.

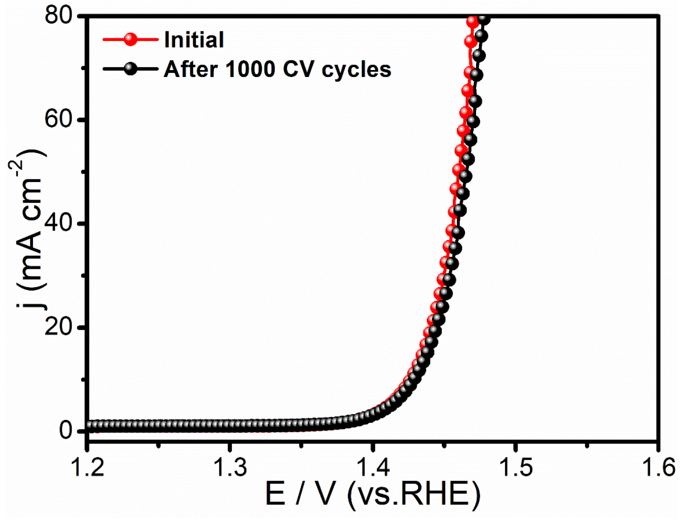

Figure S28. The accelerated degradation measurements of MD-RuO_2-BN for OER.

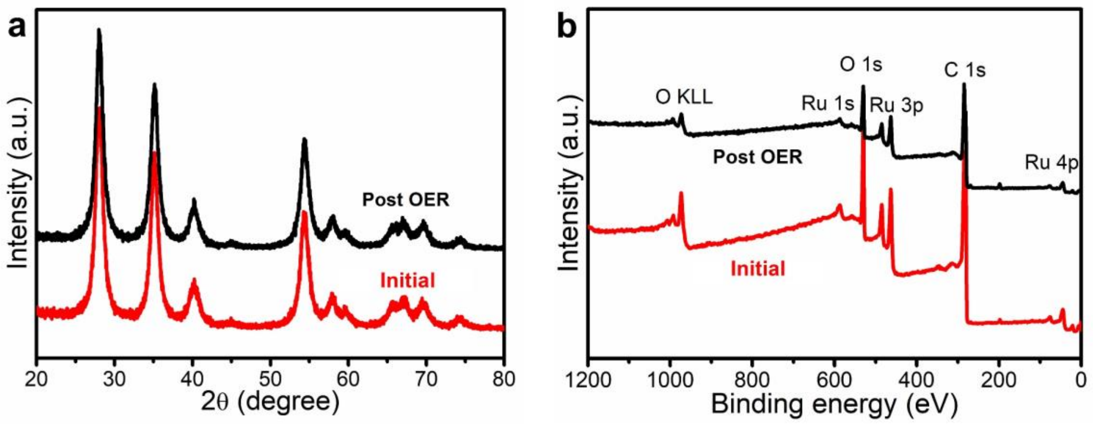

Figure S29. (a) XRD pattern and (b) XPS survey of MD-RuO_2-BN before and after OER electrolysis in 0.5 M H_2SO_4.

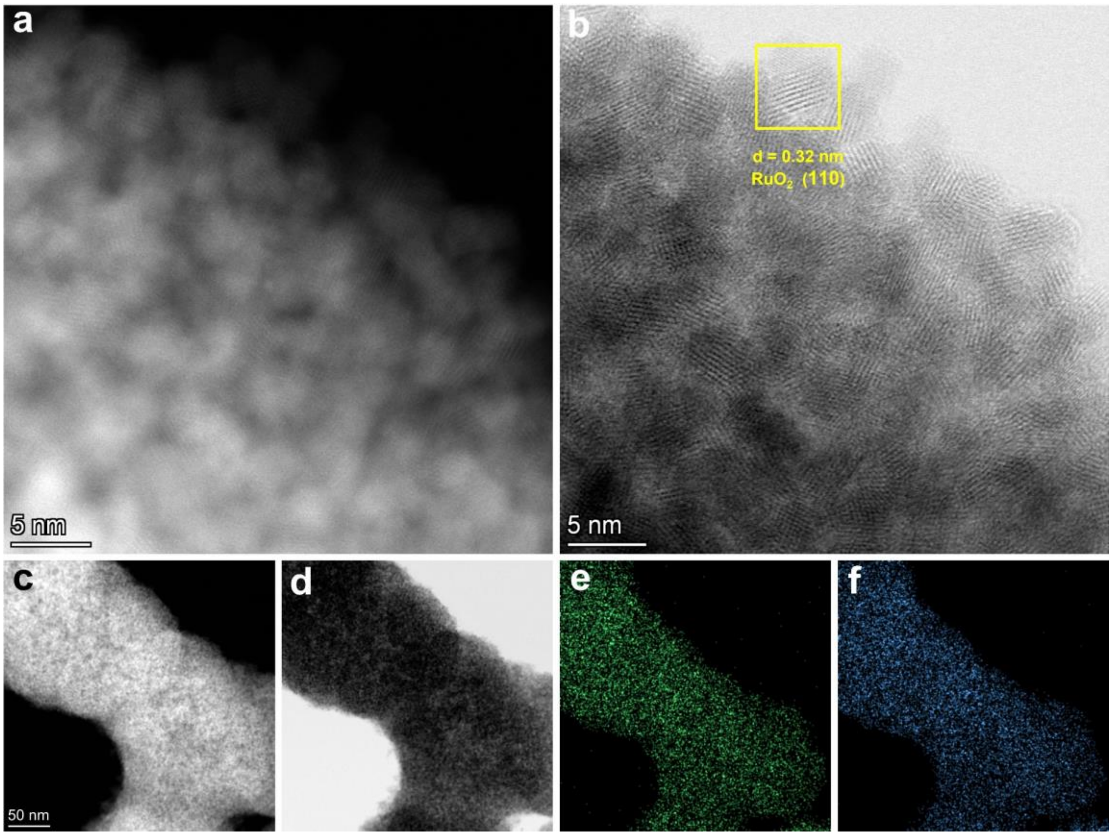

Figure S30. STEM images of MD-RuO_2-BN after OER electrolysis in 0.5 M H_2SO_4. (a) STEM-HAADF image. (b) Corresponding STEM-BF image. (c-d) STEM images and corresponding EDX elemental maps for Ru (e) and O (f).

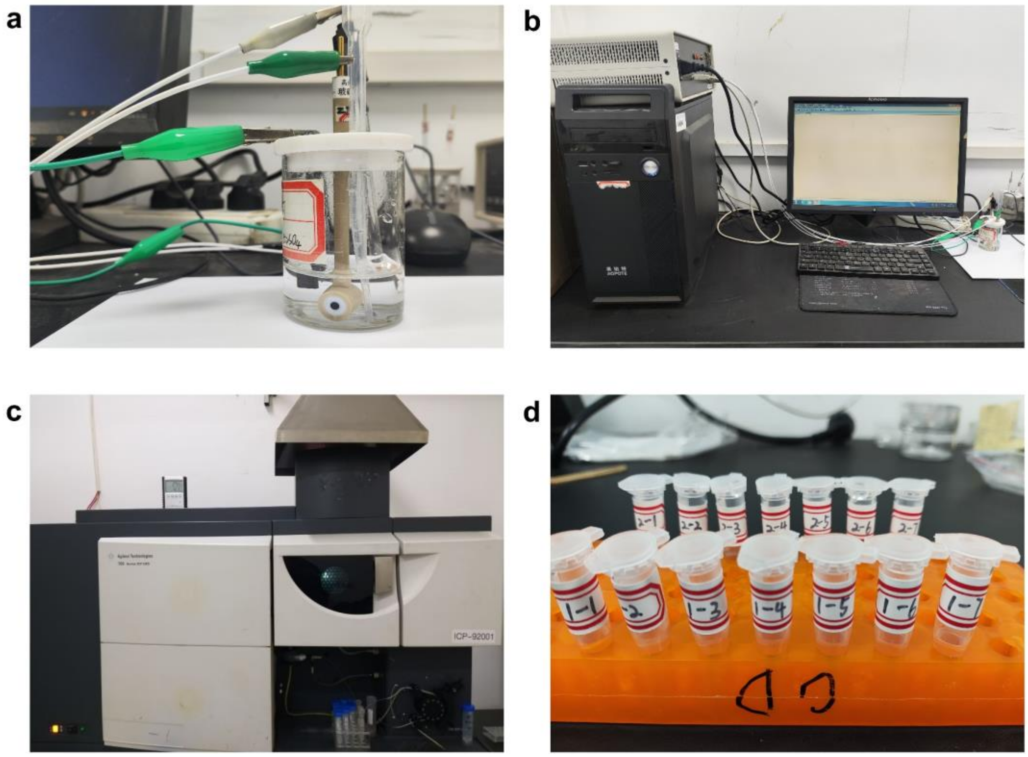

Figure S31. The experimental set-up of ICP-OES. Photographs of (a) the conventional three-electrode system, (b) the electrochemical testing system, (c) the ICP-OES (700 Series, Agilent Technologies). (d) Photographs of electrolytes collected by step-by-step (1-1~1-7 for MD-RuO_2-BN; 2-1~2-7 for K-RuO_2), the time interval was 1 day (24 h), a total of 7 days.

Supplementary Note: the chronopotentiometric measurements of MD-RuO_2-BN and K-RuO_2 (mass loading of 0.1 mg on glassy carbon electrode) for 7 days at a constant current density of 10 mA cm^{-2} was conducted in a conventional three-electrode system (Figure S31a) using a CHI 660E electrochemical analyzer (CHI Instruments, Shanghai, China, Figure S31b). Based on regular monitoring (the time interval was 24 h) the Ru concentration in 0.5 M H_2SO_4 electrolyte (25 mL) by ICP-OES (Agilent ICPOES700, Figure S31c), the dissolution of Ru was gradually collected (Figure S31d).

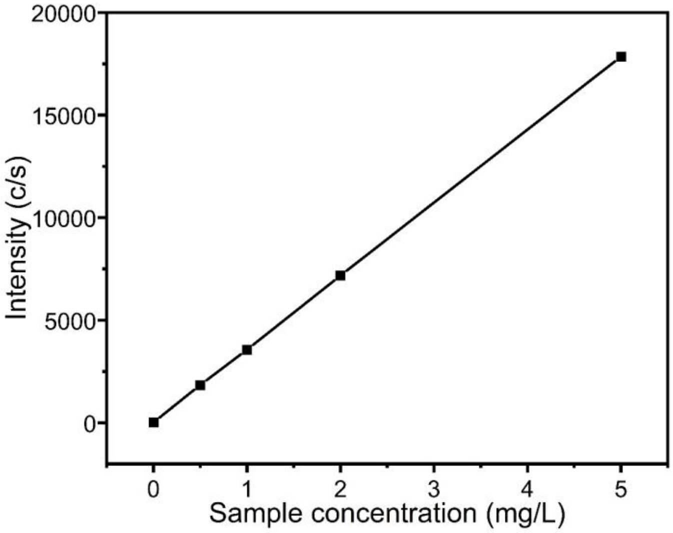

Figure S32. The obtained standardization curve of Ru standard solution. For ICP-OES studies, we first taken 0, 0.25, 0.5, 1.0, 2.5 mL of 100 mg/L Ru standard solution, respectively, into a 50 mL volumetric bottle, then add 5% dilute nitric acid to steady volume to 50 mL and shake well to obtain above standardization curve.

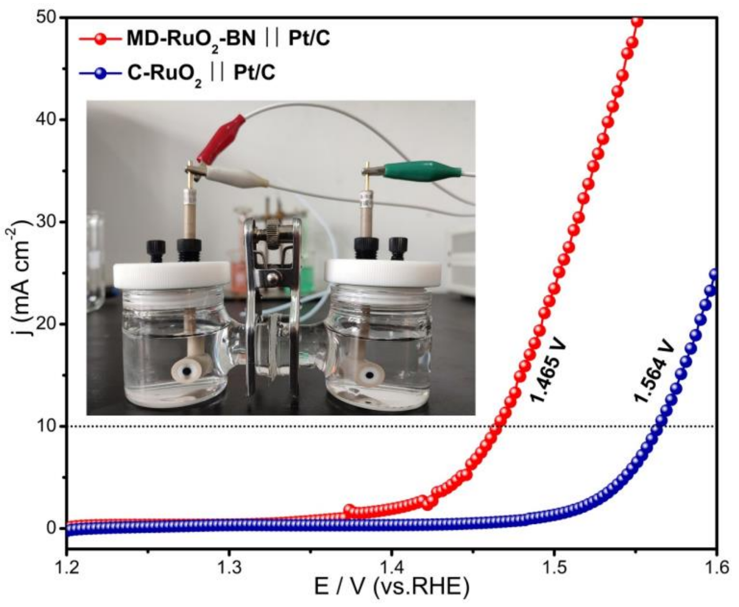

Figure S33. MD-RuO_2-BN || Pt/C and C-RuO_2 || Pt/C toward overall water splitting. Inset: the device of water splitting.

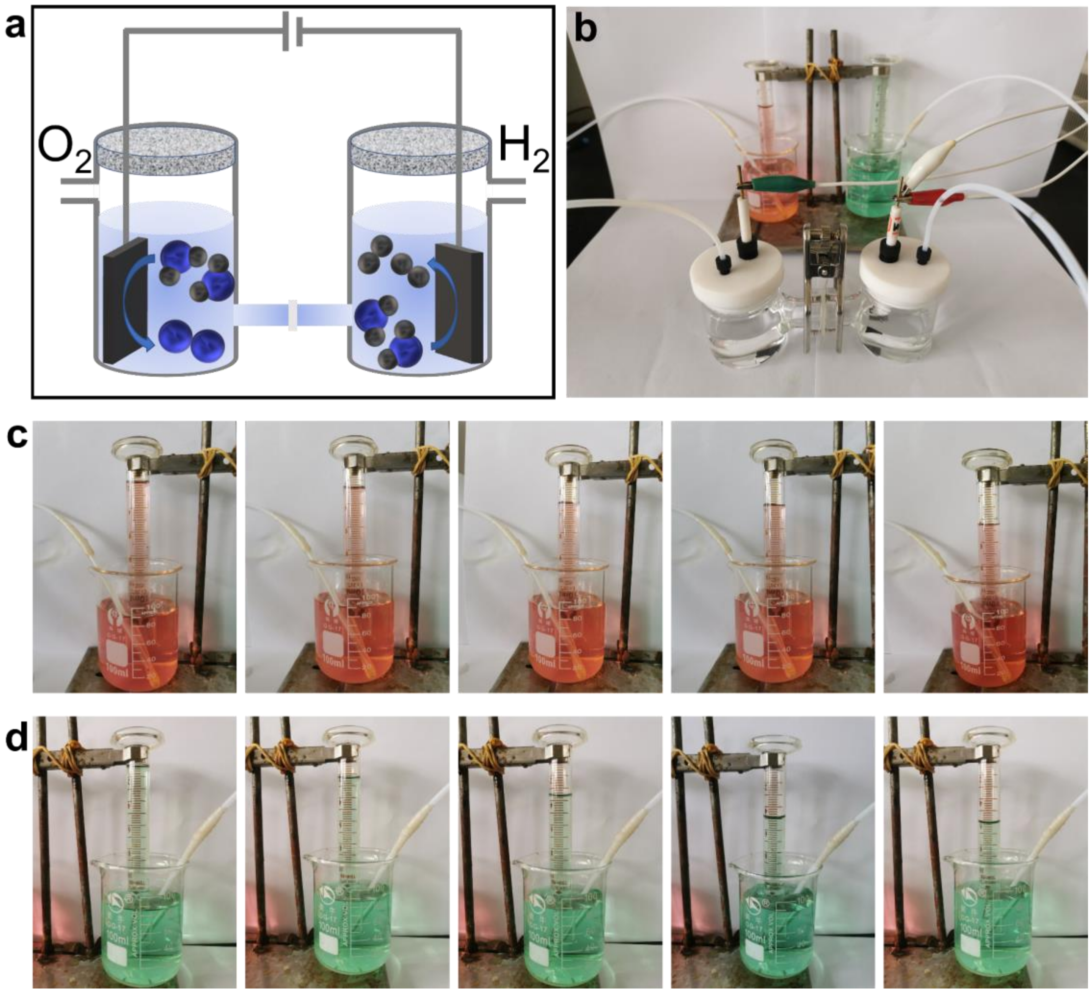

Figure S34. (a) Scheme of the two-electrode cell. (b) Gas collection device of water splitting. Photographs of oxygen (c) and hydrogen (d) collected at different times.

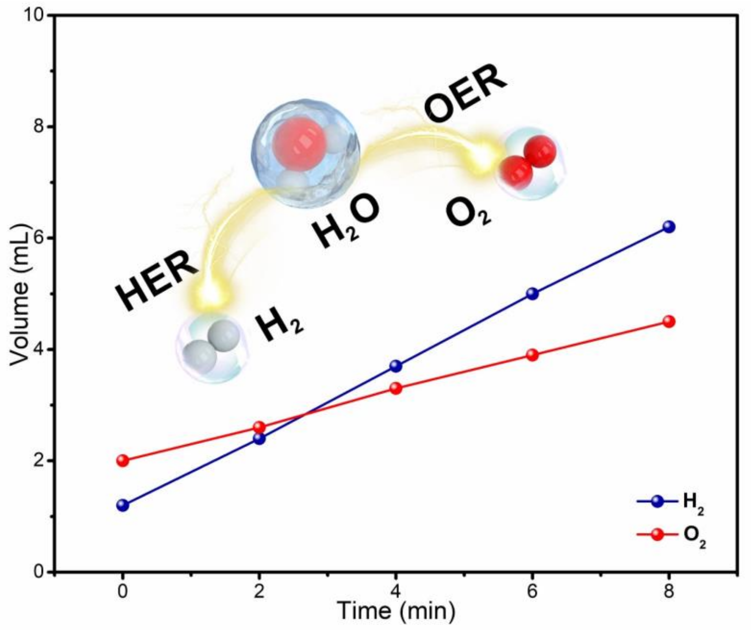

Figure S35. Amount of H_2 and O_2 in water electrolysis as a function of time.

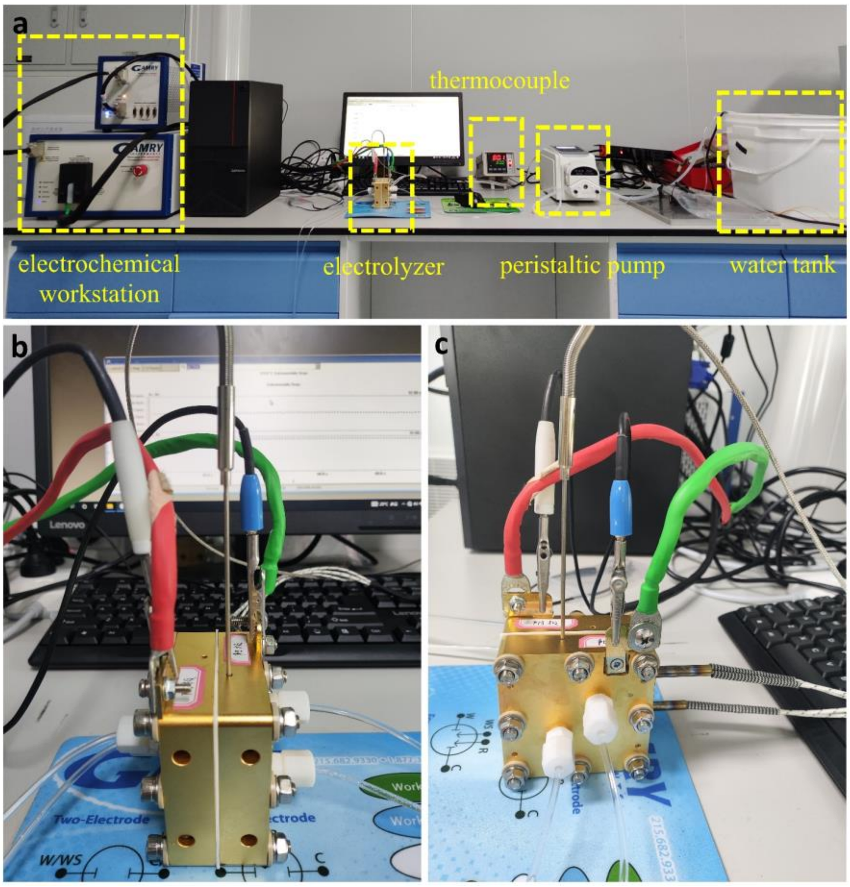

Figure S36. (a) Photographs of the PEMWE testing system, including electrochemical workstation, electrolyzer, thermocouple, peristaltic pump and water tank. (b, c) Enlarged close-up of core component electrolyzer from different perspectives.

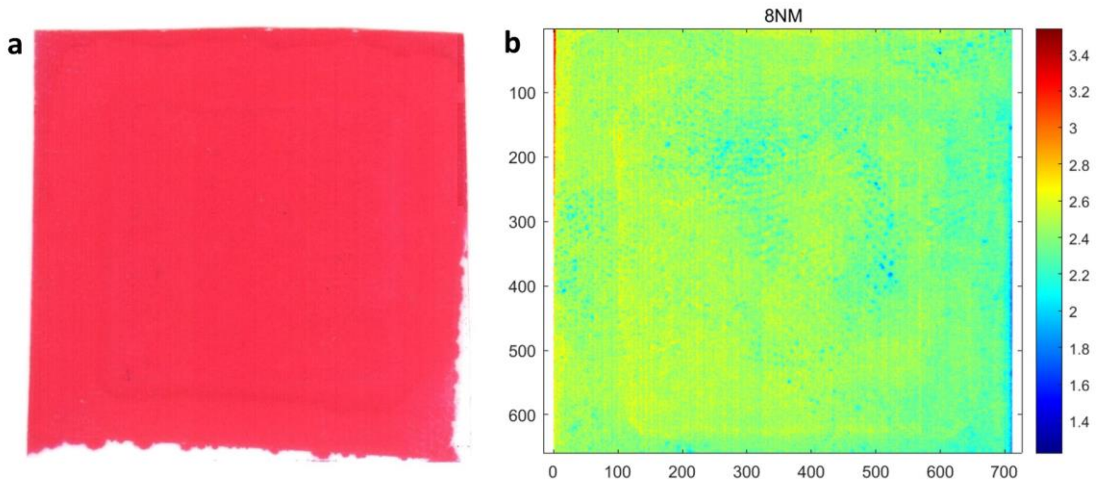

Figure S37. (a) Photograph of pressure-sensitive paper. (b) Surface stress distribution nephogram of PTL and pressure-sensitive paper.

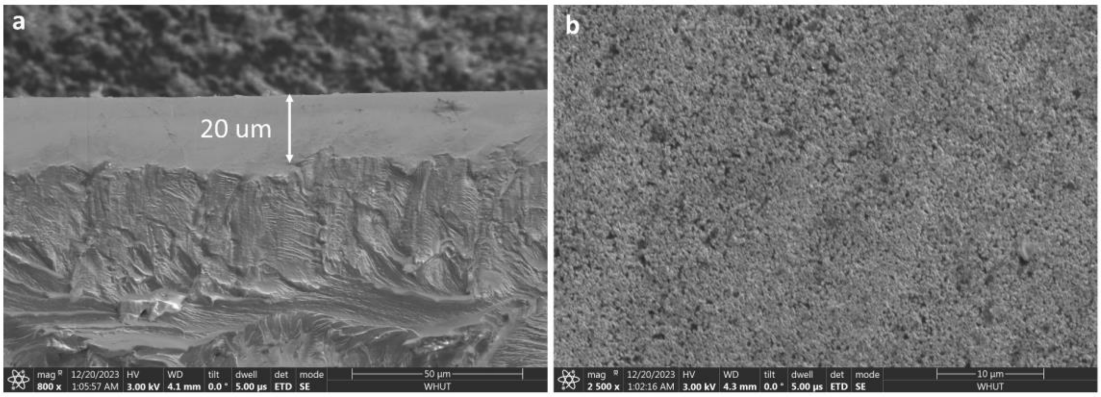

Figure S38. (a) Cross-sectional and (b) planar SEM images of MD-RuO_2-BN coated membrane.

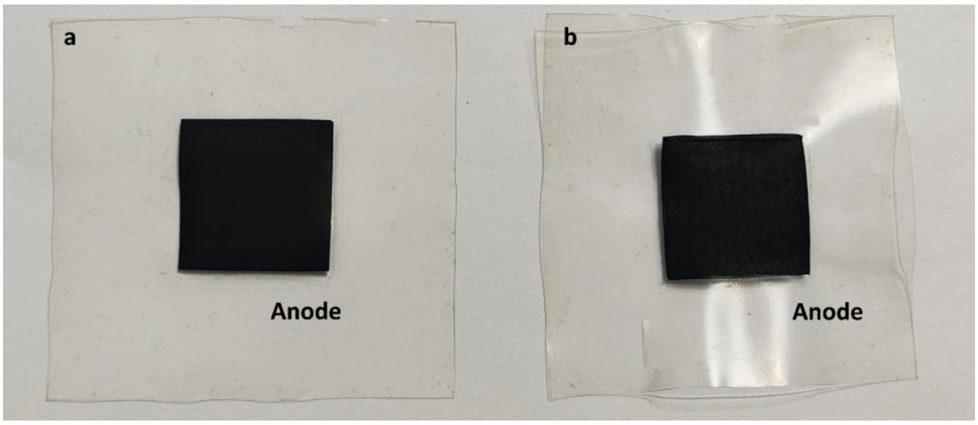

Figure S39. Photos of the electrodes before (a) and after (b) PEMWE testing.

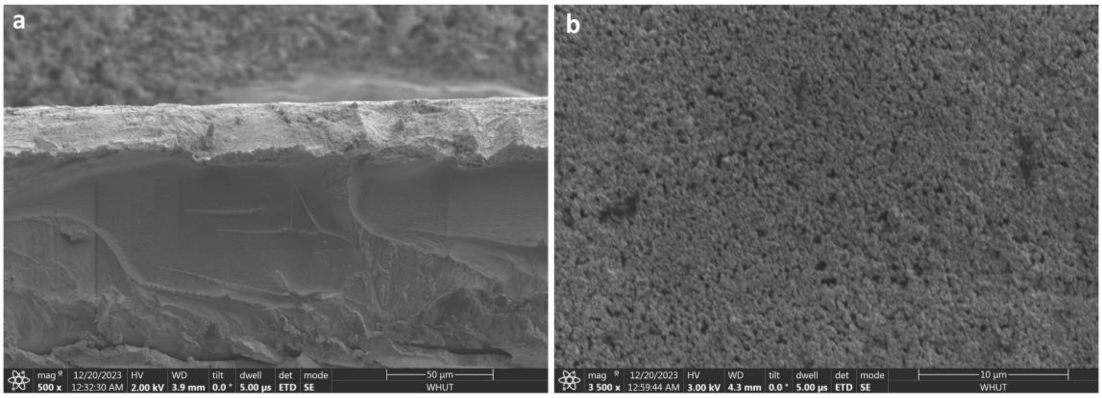

Figure S40. (a) Cross-sectional and (b) planar SEM images of MD-RuO_2-BN coated membrane after PEMWE testing.

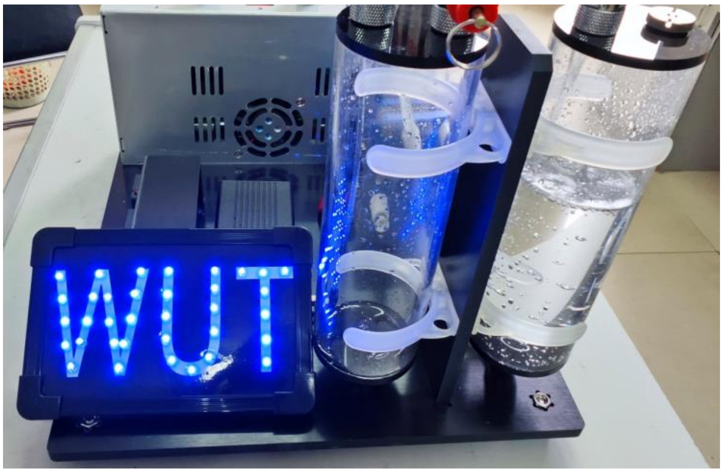

Figure S41. Demonstrate the use of hydrogen produced from PEMWE to drive hydrogen fuel cell.

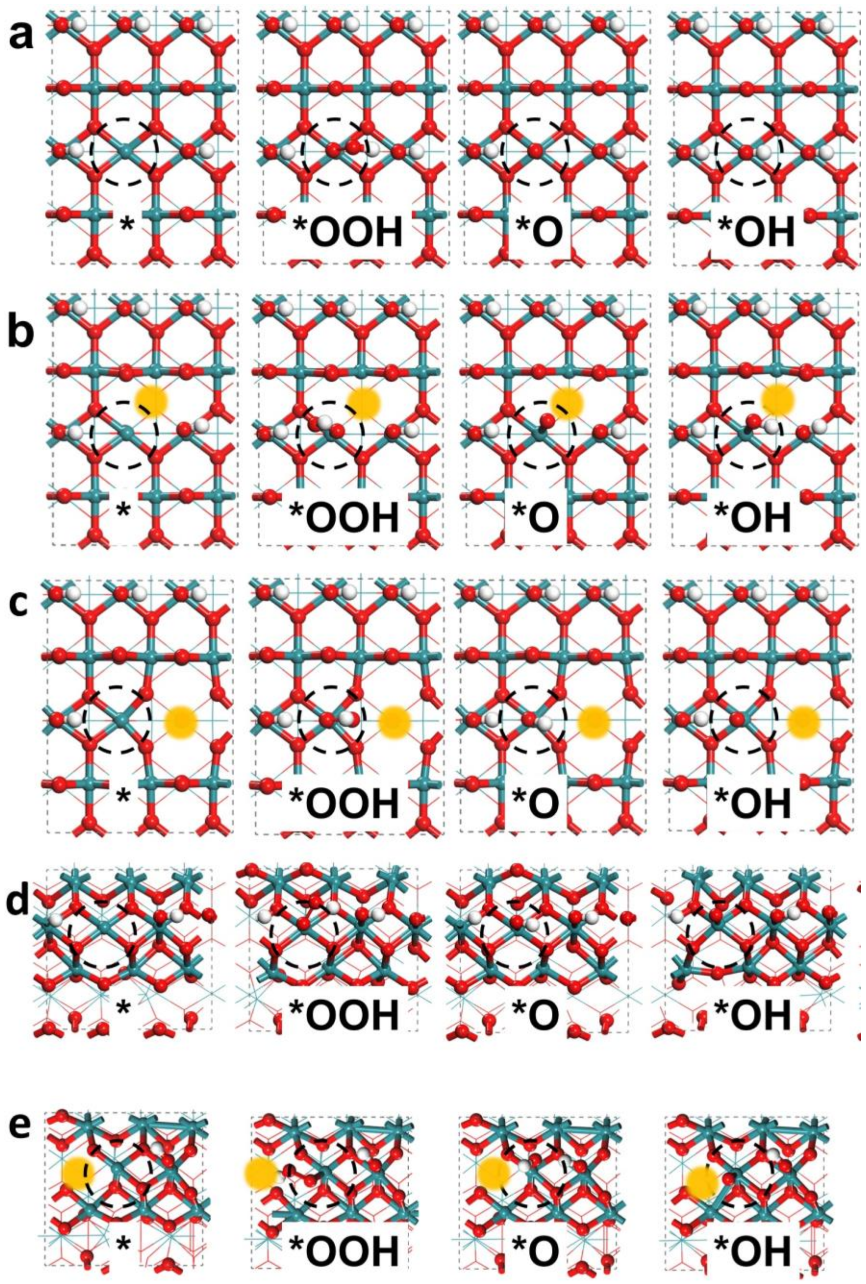

Figure S42. Top views of the *OOH, *OH, *O adsorption configuration on routine Ru sites for RuO_2 (a), RuO_2-V_O (b), RuO_2-V_{Ru} (c), RuO_2-T (d), and RuO_2-T-$V_{Ru, O}$ (e). The white, red, and indigo balls represent the H, O, and Ru atoms, respectively. The black circles and yellow highlights denote the active sites and vacancy, respectively.

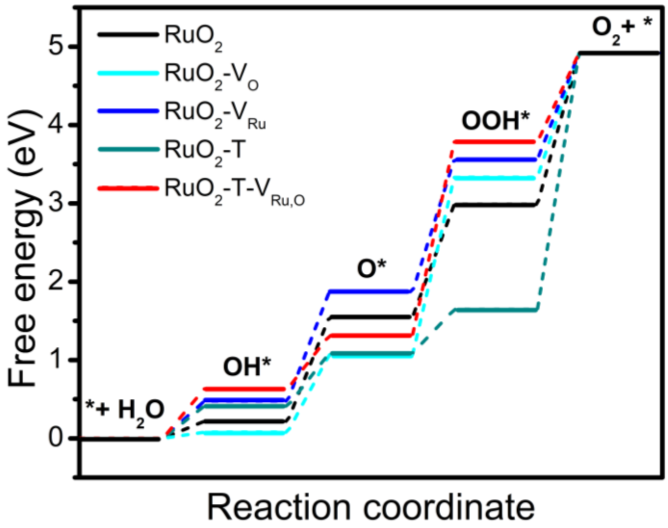

Figure S43. The free energy of OER critical intermediates including OH*, O*, OOH* on routine Ru site for RuO_2, RuO_2-V_O, RuO_2-V_{Ru}, RuO_2-T, and RuO_2-T-$V_{Ru, O}$.

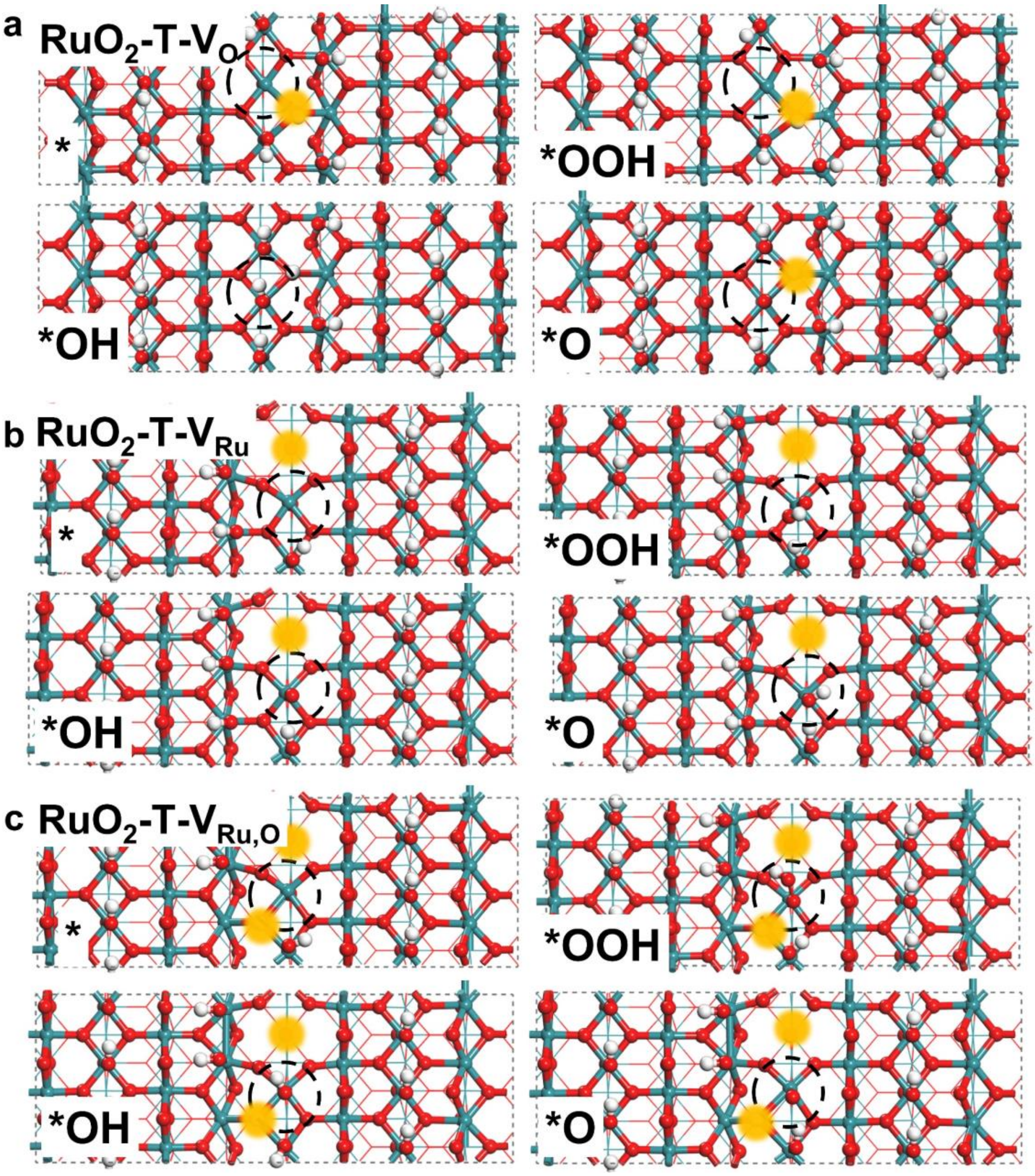

Figure S44. Top views of the *OOH, *OH, *O adsorption configuration on TB-Ru sites for RuO_2-T-V_O (a), RuO_2-T-V_{Ru} (b), and RuO_2-T-$V_{Ru,\ O}$ (c). The white, red, and indigo balls represent the H, O, and Ru atoms, respectively. The black circles and yellow highlights denote the active sites and vacancy, respectively.

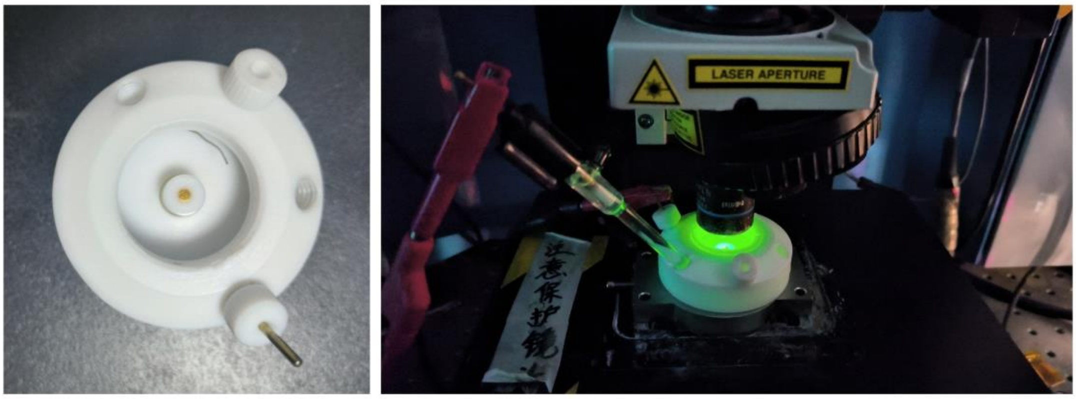

Figure S45. Photographs of experimental set-up of in-situ Raman (Horiba LabRAM HR Evolution).

Table S1. Comparison of OER performance of MD-RuO_2-BN with recently reported Ru-based electrocatalysts at 10 mA cm^{-2} in acidic media.

Catalyst	Electrolyte	Overpotential (mV)	reference
MD-RuO_2-BN	0.5 M H_2SO_4	196	This work
C-RuO_2	0.5 M H_2SO_4	305	This work
Ni-RuO_2	0.1 M $HClO_4$	214	[1]
PtCo-RuO_2/C	0.1 M $HClO_4$	213	[2]
Sr-Ru-Ir ternary oxide	0.5 M H_2SO_4	190	[3]
a/c-RuO_2	0.1 M $HClO_4$	205	[4]
RuO_2 NSs	0.5 M H_2SO_4	199	[5]
$Li_{0.52}RuO_2$	0.5 M H_2SO_4	156	[6]
RuO_2-NS/CF	0.5 M H_2SO_4	212	[7]
C-RuO_2-RuSe	0.5 M H_2SO_4	212	[8]
$Ru_1Ir_1O_x$	0.5 M H_2SO_4	204	[9]
Cu-doped RuO_2	0.5 M H_2SO_4	188	[10]
Ru_3MoCeO_x	0.5 M H_2SO_4	164	[11]
IrRu@Te	0.5 M H_2SO_4	220	[12]
Ru-N-C	0.5 M H_2SO_4	267	[13]
$RuNi_2$@G-250	0.5 M H_2SO_4	227	[14]
Ruthenate NSs	0.1 M $HClO_4$	255	[15]

Table S2. ICP investigation at regular intervals (24 h) during OER processes catalyzed by MD-RuO_2-BN and K-RuO_2, respectively.

Samples	Measured element	Content (mg/L)	Dissolution rate
MD-RuO_2-BN-1	Ru	0.10	3.29 %
MD-RuO_2-BN-2	Ru	0.11	3.62 %
MD-RuO_2-BN-3	Ru	0.12	3.95 %
MD-RuO_2-BN-4	Ru	0.15	4.93 %
MD-RuO_2-BN-5	Ru	0.15	4.93 %
MD-RuO_2-BN-6	Ru	0.16	5.26 %
MD-RuO_2-BN-7	Ru	0.17	5.59 %
K-RuO_2-1	Ru	0.15	4.93 %
K-RuO_2-2	Ru	0.17	5.59 %
K-RuO_2-3	Ru	0.19	6.25 %
K-RuO_2-4	Ru	0.19	6.25 %
K-RuO_2-5	Ru	0.20	6.58 %
K-RuO_2-6	Ru	0.22	7.24 %
K-RuO_2-7	Ru	0.24	7.89 %

Supplementary Note: The chronopotentiometric measurements were performed in a conventional three-electrode system at room temperature. The OER current density was set at 10 mA cm^{-2}. The catalyst loading was all 0.1 mg. The electrolyte was 25 mL of 0.5 M H_2SO_4. The time interval for collection was 24 h.

References:

1. Non-iridium-based electrocatalyst for durable acidic oxygen evolution reaction in proton exchange membrane water electrolysis. **Nature Materials**. 2022. DOI: 10.1038/s41563-022-01380-5.
2. Safeguarding the RuO_2 phase against lattice oxygen oxidation during acidic water electrooxidation. **Energy & Environmental Science**. 2021. DOI: 10.1039/d1ee02636d.
3. Stabilizing Highly Active Ru Sites by Suppressing Lattice Oxygen Participation in Acidic Water Oxidation. J**. Am. Chem. Soc.** 2021, 143, 6482-6490.
4. Sodium-Decorated Amorphous/Crystalline RuO_2 with Rich Oxygen Vacancies: A Robust pH-Universal Oxygen Evolution Electrocatalyst. **Angew. Chem. Int. Ed.** 2021, 60, 2-11.
5. Boosting the oxygen evolution reaction using defect-rich ultra-thin ruthenium oxide nanosheets in acidic media. **Energy & Environmental Science**. 2020. DOI: 10.1039/d0ee01960g
6. RuO_2 electronic structure and lattice strain dual engineering for enhanced acidic oxygen evolution reaction performance. **Nat. Commun**. 2022. DOI: 10.1038/s41467-022-31468-0.
7. Structure Engineering Defective and Mass Transfer-Enhanced RuO_2 Nanosheets for Proton Exchange Membrane Water Electrolyzer. **Nano Energy**. 2021. DOI: 10.1016/j.nanoen.2021.106276.
8. Exceptionally active and stable RuO_2 with interstitial carbon for water oxidation in acid. **Chem**. 2022. DOI: 10.1016/j.chempr.2022.02.003.
9. Regulating Electron Redistribution of Intermetallic Iridium Oxide by Incorporating Ru for Efficient Acidic Water Oxidation. **Adv. Energy Mater**. 2021, *11*, 2102883.
10. Assembling Ultrasmall Copper-Doped Ruthenium Oxide Nanocrystals into Hollow Porous Polyhedra: Highly Robust Electrocatalysts for Oxygen Evolution in Acidic Media. **Adv. Mater.** 2018, *30*, 1801351.
11. Tuning electron correlations of RuO_2 by co-doping of Mo and Ce for boosting

electrocatalytic water oxidation in acidic media. **Applied Catalysis B: Environmental.** 2021. DOI: 10.1016/j.apcatb.2021.120528

12. Strong electronic coupling between ultrafine iridium-ruthenium nanoclusters and conductive, acid-stable tellurium nanoparticle support for efficient and durable oxygen evolution in acidic and neutral media. **ACS Catal**. 10, 3571-3579 (2020).
13. Dynamic oxygen adsorption on single-atomic ruthenium catalyst with high performance for acidic oxygen evolution reaction. **Nat. Commun**. 10, 4849 (2019).
14. Robust interface Ru centers for high-performance acidic oxygen evolution. **Adv. Mater.** 32, 1908126 (2020).
15. Ruthenium oxide nanosheets for enhanced oxygen evolution catalysis in acidic medium. **Adv. Energy Mater**. 9, 1803795 (2019).
