# Supplementary material for: Bicontinuous RuO2 nanoreactors for acidic water oxidation
Source: Nat Commun. 2024 May 9;15:3928. doi: 10.1038/s41467-024-48372-4 (PMC11082236; doi:10.1038/s41467-024-48372-4)
Supplement: Supplementary file 3 — Description of Additional Supplementary Files [file 41467_2024_48372_MOESM3_ESM.pdf]

Description of Additional Supplementary Files

Supplementary Movie 1: Dynamic tomography reconstruction process of representative position.

Supplementary Movie 2: The rotation, filtering and ortho-slicing dynamic process of the resulting reconstructed structural units.

Supplementary Movie 3: The rotation, filtering and ortho-slicing dynamic process of the extracted cubic sub volume from the resulting reconstructed structural units.

Supplementary Movie 4: Synchronous rotation, filtering and ortho-slicing dynamic process of high contrast volume (represents for RuO2) and low contrast one (represents for interspace).

Supplementary Movie 5: Demonstrate the use of hydrogen produced from PEMWE to drive hydrogen fuel cell.
